# Cytotoxicity of hydro-alcoholic extracts of *Cucurbita* *pepo* and *Solanum nigrum* on HepG2 and CT26 cancer cell lines

**DOI:** 10.4103/0973-1296.66931

**Published:** 2010

**Authors:** M. Shokrzadeh, M. Azadbakht, N. Ahangar, A. Hashemi, S. S. Saeedi Saravi

**Affiliations:** *Department of Toxicology Pharmacology, Mazandaran University of Medical Sciences, Sari, Mazandaran Pharmaceutical Sciences Research Center, Iran*; 1 *Department of Pharmacognosy, Mazandaran University of Medical Sciences, Sari, Mazandaran Pharmaceutical Sciences Research Center, Iran*; 2 *Faculty of Pharmacy, Mazandaran University of Medical Sciences, Sari, Mazandaran Pharmaceutical Sciences Research Center, Iran*

**Keywords:** Cell lines, colonogenic assay, Cucurbita *pepo*, cytotoxicity, *Solanum nigrum*

## Abstract

Plants are used worldwide for the treatment of diseases, and novel drugs continue to be developed through research from plants. There are more than 20,000 species of plants used in traditional medicines, and these are all potential reservoirs for new drugs. *Cucurbita* *pepo* has been used in traditional folk medicine to treat cold and alleviate ache. Previous pharmacological tests have shown that it possesses antiviral, anti-inflammatory, and analgesic effects. Also, *Solanum nigrum* has been used as a diuretic and an antipyretic agent and it has also been used to cure inflammation, edema, mastitis and hepatic cancer. In this investigation, cytotoxicity of specific concentrations of hydro-alcoholic extracts of C. *pepo* and S. nigrum was studied on normal [Chinese hamster ovarian cells (CHO) and rat fibroblast] and cancer (HepG2 and CT26) cell lines. The cytotoxic effects and IC_50_ of the extracts on the selected cell lines were studied followed by colonogenic assay method. The results showed that IC_50_ of S. nigrum extract was significantly lower than that of the C. *pepo* extract on all four cell lines (*P* < 0.05). On the other hand, IC_50_ of S. nigrum extract was significantly higher than the extract of *Taxus baccata* and Cisplatin, herbal and chemical control positive anticancer compounds, respectively, on all four cell lines (*P* < 0.05). As a result, it is concluded that the extract of S. nigrum has almost similar cytotoxicity to the extract of T. baccata on cancer cells.

## INTRODUCTION

Plants are used worldwide for the treatment of diseases, and novel drugs continue to be developed through research from plants. There are more than 20,000 species of plants used in traditional medicines, and these are all potential reservoirs for new drugs.[1] With the advance in modern medicine and drug research, chemical synthesis has replaced plants as the primary source of medicinal agents in industrialized countries. In developing countries, the majority of the world’s population cannot afford pharmaceutical drugs and use their own plant-based indigenous medicines. Traditional medicinal plants have received considerable attention because their bioactive components may lead to new drug discoveries.[2–10]

*Solanum nigrum* Linn. (Solanaceae), commonly known as “Black nightshade,” has been extensively used in traditional medicine in different parts of world to cure liver disorders, chronic skin ailments (Psoriasis and ringworm), inflammatory conditions, painful periods, fevers, diarrhea, eye diseases, hydrophobia, seizure, etc.[11–14] Also, the fruits are believed to have anti-tumor properties, although the mechanism for the activity remains to be elucidated.

*Cucurbita pepo* (Cucurbitaceae), commonly known as “pumpkin,” distributed in different regions of world, has been employed in folk medicine to treat colds, alleviate aches, benign prostatic hypertrophy (BPH), etc. Previous pharmacological tests have shown that it possesses antibacterial, antiviral, anti-inflammatory and analgesic effects.[15,16] Taxol is an intense anti-tumor compound extracted from the plants belonged to *Taxus* species, such as *Taxus baccata*. However, difficulty in obtaining this compound from yew trees has limited its clinical use.[17]

In this investigation, cytotoxic effects and IC_50_ of specific concentrations of hydro-alcoholic extracts of fruits of *S. nigrum* and leaves of *C.pepo* were compared with hydro-alcoholic extract of bark of T. baccata and Cisplatin, well-known herbal and chemical anticancer compounds, respectively, on normal [Chinese hamster ovarian cells (CHO) and rat fibroblast] and cancer (HepG2 and CT26) cell lines.

## MATERIALS AND METHODS

### Plant material

Leaves of *C. pepo* and bark of *T. baccata* were collected from the northern regions of Iran (Neka city in Mazandaran province) in August 2007. Also, fruits of *S. nigrum* were collected from downtown of Isfahan, Isfahan province. Botanical identification was confirmed by morphologic characteristics at Department of Pharmacognosy, Sari Faculty of Pharmacy.

### Extraction and isolation

A measured quantity of 50 g of dried and powdered parts of each plant was chopped and soaked in 500 ml of ethanol (50% v/v) for 48 h and then percolated (10 drops/min).[18,19] The hydro-alcoholic extracts (1:10) were separately concentrated over a rotary vacuum evaporator and specific concentrations of the hydro-alcoholic extracts (5, 25, 50, 100, 150 µg/ml) were prepared using phosphate buffer (pH = 7.4).

### Cell lines

Normal CHO, normal rat fibroblast, HepG2 (human hepatocarcinoma) and CT26 (human colon carcinoma) cell lines were purchased from Pasture Institute of Iran in Tehran.

The completed media were sterilized by 0.22 µm microbiological filters and kept at 4°C before use.

### Colonogenic assay

In colonogenic assay, 50 µl of DMEM/F12 including 500–700 cells was added to three wells of six well/plates for each concentration of the extracts and Cisplatin. Then, they were incubated for 48 h. After incubation, the cell lines were exposed to 50 µl of 0 (phosphate buffer), 5, 25, 50, 100, 150 µg/ml of hydro-alcoholic extracts of *C. pepo*, *S. nigrum* and *T. baccata*, and 50 µl of 0, 2.5, 5, 10, 25 µg/ml of Cisplatin for 2 h, and then washed using sterile normal saline 0.09%. Then, 4 ml fresh culture media was added to the wells and incubated for 7 days. After this period, the contents of wells were excluded; the cells were fixed with formalin 9%, and dyed with trypan blue 4% (w/v) for 20 min. Then, trypan blue was excluded, and the six well/plates were washed using sterile normal saline 0.09%. At the end, the dyed colonies were counted using a light microscope.[19–21]

### Statistical analysis

Prism ver.3 Software was used to perform statistical tests. One way analysis of variance (ANOVA) followed by Tukey test were used to determine the differences among the groups (*P* < 0.05).

## RESULTS

The results showed that the extract of *S. nigrum* possesses lower IC_50_ compared to that of the *C. pepo* extract on all four normal and cancer cell lines (*P* < 0.05). But IC_50_ of the *S. nigrum* extract was significantly higher than that of the extract of *T. baccata* and Cisplatin on all four normal and cancer cell lines (*P* < 0.05) [Table 1]. The lower IC_50_ represents the higher potency of a compound to inhibit the growth of cells and cause toxicity and death of cells.

**Table 1:** The evaluated IC_50_ of *C. pepo, S. nigrum* and *T. baccata* extracts, and Cisplatin on the selected normal and cancer cell lines

Compound	Cell lines			
	Cancer cell line		Normal cell line	
	HepG2 (1C_50_^a^ ± SD)*	CT26 (1C_50_^a^ ± SD)*	CHO (1C_50_^a^ ± SD)*	Fibroblast (1C_50_^a^ ± SD)*
*C. pepo* (leaves)	132.6 ± 4.3	167.2 ± 6.3	239.2 ± 10.3	241.4 ± 9.6
*S. nigrum* (fruits)	56.4 ± 9.3	77.6 ± 1.2	98.2 ± 2.1	102.3 ± 6.1
*T. baccata* (bark)	16.6 ± 0.8	18.7 ± 0.3	20.4 ± 1.2	19.1 ± 1.1
Cisplatin	0.07 ±0.87	1.2 ± 0.03	5.5 ± 0.21	1.6 ± 0.21

a µg/ml

* *P* < 0.05

**Figure 1:**
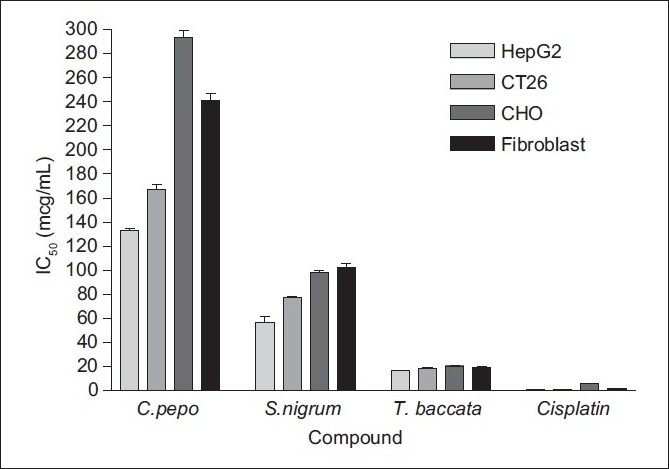
The evaluated IC_50_ of *C. pepo, S. nigrum* and *T. baccata* extracts, and Cisplatin on the selected normal and cancer cell lines

Comparison of the evaluated IC_50_ of the *S. nigrum* and *C. pepo* extracts with that of the *T. baccata* extract and Cisplatin on normal and cancer cell lines are shown in Figure 1. However, the lowest and highest IC_50_ values were related to Cisplatin and hydro-alcoholic extract of *C. pepo* in all cell lines.

IC_50_ of the compounds on the four cell lines increased in the following rank order: Cisplatin < *T. baccata* < *S. nigrum* < *C. pepo*. The IC_50_ of Cisplatin on the four cell lines decreased in the following rank order of cells: CHO > fibroblast > CT26 > HepG2. Also, the IC_50_ of hydro-alcoholic extract of *T. baccata* on the four cell lines decreased in the following rank order of cells: CHO > fibroblast > CT26 > HepG2.

On the other hand, the IC_50_ of hydro-alcoholic extract of *S. nigrum* on the four cell lines increased in the following rank order of cells: fibroblast < CHO < CT26 < HepG2. As a result, the highest and lowest cytotoxicity of the *S. nigrum* extract was related to HepG2 (IC_50_ = 56.4 ± 9.3 µg/ml) and CHO (IC_50_ = 102.3 ± 6.1 µg/ml) cell lines.

The IC_50_ of hydro-alcoholic extract of *C. pepo* on the four cell lines increased in the following rank order of cells: CHO < fibroblast < CT26 < HepG2. Also, the highest and lowest cytotoxicity of the *C. pepo* extract was related to HepG2 (IC_50_ = 132.6 ± 4.3 µg/ml) and fibroblast (IC_50_ = 293.2 ± 10.3 µg/ml) cell lines.

## DISCUSSION

For many years, plants have been used for treating different types of diseases in human beings. As per the World Health Organization (WHO) calculation, about 80% of the world’s population presently uses medicinal herbal drugs for their primary health care.[22,23] Cancer is one of the main health problems of the world′s population. Current chemotherapeutic drugs are not useful in all cases and have severe side effects on human health. As a result, a search for other alternatives, preferably natural products, seems necessary and beneficial. This leaves an open door for new and better compounds. In continuation of our search for substances of plant origin with pharmacological effects, we have isolated and screened the plants *S. nigrum* and *C. pepo* for their cytotoxic activities.

According to the results, IC_50_ of the *S. nigrum* and *C. pepo* extracts, and *T. baccata* extract and Cisplatin, as herbal and chemical control positive compounds, respectively, on normal cell lines were higher than that on cancer cell lines. This difference can result from dysfunction of cellular organisms following cancer incidence which causes higher rate of proliferation and increased cellular intake. Also, defensive disorders and effusion insufficiency to escape toxic substances from cells can lead to a lower necessity of the amounts of cytotoxic compounds to inhibit the growth of cancer cells, in comparison with normal cells.[19–21]

A study carried out by Patel *et al*., evaluated that methanolic extract of *S. nigrum* fruits has potential activity on HeLa cell and lesser effect on Vero cell. So, the drug has considerable anticancer activity on cervical cancer.[24] Wang *et al*. showed antitumor effects of methanolic extract of *S. nigrum* fruits on U266 cells.[25] During Wang *et al*.’s search for new anti-tumor agents, the ethanolic extract of fruits of *C. pepo* was observed to exhibit a significant dose-dependent inhibitory effect against HeLa cell growth.[26]

In our study, MTT[3-(4,5-dimethylthiazol-2-yl)-2,5-diphenyltetrazolium bromide] assay shows considerable activity of *S. nigrum* on HepG2 cell and little beat effect on CT26 cell. Also, the viability percents of the cells exposed to the extract of *S. nigrum* and similarity of IC_50_ of this extract with the *T. baccata* extract, a common natural anticancer product, allows us to conclude that extracts of different parts of *S. nigrum* are good candidates for further studies of activity-monitored fractionation to identify their active components. Our results further support the idea that these medicinal plants can be promising sources of potential anticancer agents to treat or control a variety of cancers such as liver and colon cancers. In addition, the results of this study can form the basis on which the researchers can conduct cytotoxic studies on different cancer cells and find the mechanisms of cytotoxicity of the plants.

## References

[1] Hamamouchi M (2002). Medicinal plants in Morocco: Traditional use, marketing, and strategies for conservation and increasing value. Esperance Med.

[2] Huang CH, Kingston DG, Magri NF, Samaranayake G, Boettner FE (1986). New taxanes from *Taxus* brevifolia. J Nat Prod.

[3] Van Uden W, Homan B, Woerdenbag HJ, Pras N, Malingre TM, Wichers HJ (1992). Isolation, purification, and cytotoxicity of 5-methoxypodophyllotoxin, a lignan from a root culture of Linum flavum. J Nat Prod.

[4] Prasain JK, Stefanowicz P, Kiyota T, Habeichi F, Konishi Y (2001). Taxines from the needles of *Taxus wallichiana*. Phytochem.

[5] Dehkordi AJ, Emami SA, Saeidi M, Sadeghi H (2004). Cytotoxicologic Studies of the Extracts of Iranian *Juniperus Sabina* and Platycladus orientalis on Cancer Cells. J Res Med Sci.

[6] Kim JS (2006). Compositions for inducing secretion of insulin-like growth factor-1.

[7] Limem-Ben Amor I, Boubaker J, Ben Sgaier M, Skandrani I, Bhouri W, Neffati A (2009). Phytochemistry and biological activities of Phlomis species. J Ethnopharmacol.

[8] Ebrahimzadeh MA, Mahmoudi M, Karami M, Saeedi Saravi SS, Ahmadi AH, Salimi E (2007). Separation of active and toxic portions in Sambucus ebulus. Pakistan J Biol Sci.

[9] Saeedi Saravi SS, Shokrzadeh M (2010). The chemistry, pharmacology and clinical properties of Sambucus ebulus: A review. J Med Plant Res.

[10] Saeedi Saravi SS, Shokrzadeh M (2008). Histopathological and Biochemical Disorders Following Administration of Sambucus ebulus Extract on Mice and Rats and Preventive Effects of Vitamins C and E on Renal and Hepatic Disorders. Phcog Mag.

[11] Kritikar KR, Basu BD (1935). Indian Medicinal Plants.

[12] Jainu M, Shyamala Devi CS (2006). Antiulcerogenic and ulcer healing effects of *Solanum nigrum* (L.) on experimental ulcer models: Possible mechanism for the inhibition of acid formation. J Ethnopharmacol.

[13] Lin HM, Tseng HC, Wang CJ, Lin JJ, Lo CW, Chou FP (2008). Hepatoprotective effects of *Solanum nigrum* Linn extract against CCl4- induced oxidative damage in rats. Chem Biol Interact.

[14] Wannang NN, Anuka JJ, Kwanashie HO, Gyang SS, Auta A (2008). Anti-seizure activity of the aqueous leaf extract of *Solanum nigrum* linn (solanaceae) in experimental animals. African Health Sci.

[15] Wang DA, Pan HU, Deng XM, Xiang H, Gao HY, Cai H (2007). Cucurbitane and hexanorcucurbitane glycosides from the fruits of *Cucurbita* *pepo* cv dayangua. J Asian Nat Product Res.

[16] Hegi G (1979). Illustrierte Flora von Mitteleuropa. Band 6, Teil 2.

[17] Bonfill M, Expüsito O, Onrubia M, Jané A, Cusidü RM, Palazün J (2007). Effect of external factors on the production of taxol and other taxanes in cell cultures of *Taxus* baccata. J Biotechnol.

[18] Prasad SB, A (1994). Giri. Antitumor effect of Cisplatin against murine ascites Dalton’s lymphoma. Indian J Exp Biol.

[19] Shokrzadeh M, Azadbakht M, Ahangar N, Naderi H, Saeedi Saravi SS (2010). Comparison of Cytotoxic Effects of Juniperus sabina and Zataria multiflora Extracts With *Taxus* baccata Extract and Cisplatin on Normal and Cancer Cell Lines. Phcog Mag.

[20] Shokrzadeh M, Saeedi Saravi SS, Mirzayi M (2009). Cytotoxic Effects of Ethyl Acetate Extract of Sambucus ebulus Compared With Etoposide on Normal and Cancer Cell Lines. Phcog Mag.

[21] Shokrzadeh M, Azadbakht M, Ahangar N, Naderi H, Saeedi Saravi SS (2009). Cytotoxic effects of hydroalcoholic extract of Juniperus sabina compared with hydroalcoholic extract of *Taxus* baccata and Cisplatin on normal and cancer cell lines. Planta Med.

[22] (2003). WHO. Diet, Nutrition and the Prevention of Chronic Diseases.

[23] Etkin NL, Hausa A (1981). Herbal Pharmacopoeia: Biomedical Evaluation of Commonly used Plant Medicines. J Ethnopharmacol.

[24] Patel S, Gheewala N, Suthar A, Shah A (2009). In-vitro cytotoxicity activity of *Solanum nigrum* extract against Hela cell line and Vero cell line. Int J Pharm Pharmaceut Sci.

[25] Wang W, Lu DP (2005). An in vitro study of cytotoxic and antineoplastic effect of *Solanum nigrum* L extract on U266. Beijing Da Xue Xue Bao.

[26] Wang DC, Pan HY, Deng XM, Xiang H, Gao HY, Cai H (2007). Cucurbitane and hexanorcucurbitane glycosides from the fruits of Cucurbita pepo cv dayangua. J Asian Nat Pro Res.

